# Identifying factors associated with substantially reduced adult height in patients with juvenile idiopathic arthritis: a retrospective cohort study

**DOI:** 10.1186/s12887-024-04855-3

**Published:** 2024-05-30

**Authors:** Hsin-Yu Chen, Ya-Chiao Hu, Yao-Hsu Yang, Bor-Luen Chiang

**Affiliations:** 1 Department of Pediatrics, Cathay General Hospital Hsinchu Branch, Hsinchu, Taiwan; 2 Department of Pediatrics, National Taiwan University Hospital, No. 8 Chung-Shan South Road, Taipei, 100 Taiwan, ROC; 3 Graduate Institute of Clinical Medicine, College of Medicine, National Taiwan University, Taipei, Taiwan; 4 Genome and Systems Biology Degree Program, College of Life Science, National Taiwan University, Taipei, Taiwan

**Keywords:** Juvenile idiopathic arthritis, Reduced adult height, Systemic JIA

## Abstract

**Background:**

Juvenile idiopathic arthritis (JIA), an autoimmune disease affecting children or adolescents and causing joint or systemic symptoms, reportedly has a negative effect on the patients’ body height. This study aimed to identify factors attributable to substantially reduced adult height (SRAH) in JIA patients.

**Methods:**

This single-center retrospective cohort study included patients from 2009 to 2019 in Taiwan. We collected JIA patients aged > 18 years at enrollment with a definite diagnosis and undergoing regular outpatient clinic follow-up or disease remission. Target height difference (THD), defined by adult height minus mid-parental height, was calculated for each patient. The calculation results yielded two groups, of which positive THD was defined as the optimal height (OH group) and those with THD below two standardized deviations as the SRAH group. Descriptive statistics and logistic regression analysis were used to analyze the data.

**Results:**

Of 92 JIA patients, 57 and 12 were in the OH and the SRAH groups. Earlier disease onset, especially before the six-year-old, was noted in the SRAH group (*p =* 0.026). The distribution of JIA subtypes differed significantly between the two groups (*p <* 0.001); enthesis-related arthritis was the commonest subtype in the OH group, and systemic JIA was the commonest in the SRAH group. Half of the patients in the SRAH group had an active disease status at enrollment, which was higher than the OH group (50.0% vs. 21.1%, *p* = 0.066). More patients in the SRAH group had received orthopedic surgery due to JIA (25% vs. 3.5%, *p* = 0.034). Multiple logistic regression analysis showed that SRAH was independently related to systemic JIA (OR = 37.6, 95%CI 1.2-1210.5; *p* = 0.041).

**Conclusion:**

The subtype of systemic JIA, with its characteristics of early disease onset and active disease status, was the essential factor that significantly impacted adult height.

**Supplementary Information:**

The online version contains supplementary material available at 10.1186/s12887-024-04855-3.

## Background

Juvenile idiopathic arthritis (JIA), the most common cause of chronic arthritis in childhood, affects specific articular joints and results in arthritis and constitutional symptoms in adolescents or children below 16 years old. JIA is classified into seven subtypes based on onset age, number of joint involvements, associated symptoms, and serological examinations of each patient [1]. A short body height in adulthood is usually a main concern in JIA patients and their parents. The estimated prevalence of short stature in JIA ranges from around 10–40% among different JIA subtypes [2, 3]. In addition, a short body height is also more prevalent in polyarticular and systemic subtypes of JIA, with prevalence rates ranging from 10.4% in polyarticular JIA and 41.0% in systemic JIA (sJIA) [3, 4]. 

The possible mechanisms of JIA-related short body height are complicated, including the inflammatory process of the disease, poor nutrition, growth hormone deficiency, and the effects of treatments, according to previous data [4–6]. Persistent inflammation plays a critical role. Previous studies revealed that pro-inflammatory cytokines negatively affected growth plate chondrogenesis [7]. The most prominent inflammatory cytokines involved in the pathogenesis of sJIA, including TNFα, interleukin-1, and interleukin-6, can influence molecular signaling pathways, chondrocyte, osteoclast, and osteoblast activities and aggravate joint inflammation [7–12]. Silencing of cytokine effects results in more stable height growth in JIA patients; however, overactivation of these cytokines might result in poor longitudinal growth [13]. Aside from the cytokines production due to active disease, several studies revealed the influence of some medications on the height of JIA patients, such as glucocorticoids (GC) and tumor necrosis factor (TNF)-α inhibitors. Wang et al. reported that using GC for more than one year may significantly impair body height growth [14]. The TNF-α inhibitor Etanercept was found to have the potential to improve body height after introducing biological agents in recent years [15, 16]. Although many studies mentioned the factors affecting growth velocity and height percentile in childhood, they seldom discussed final adult height (AH) in JIA. In this study, we aimed to identify the factors that may contribute to substantially reduced adult height (SRAH) in patients with JIA.

## Methods

### Study populations

**Fig. 1:**
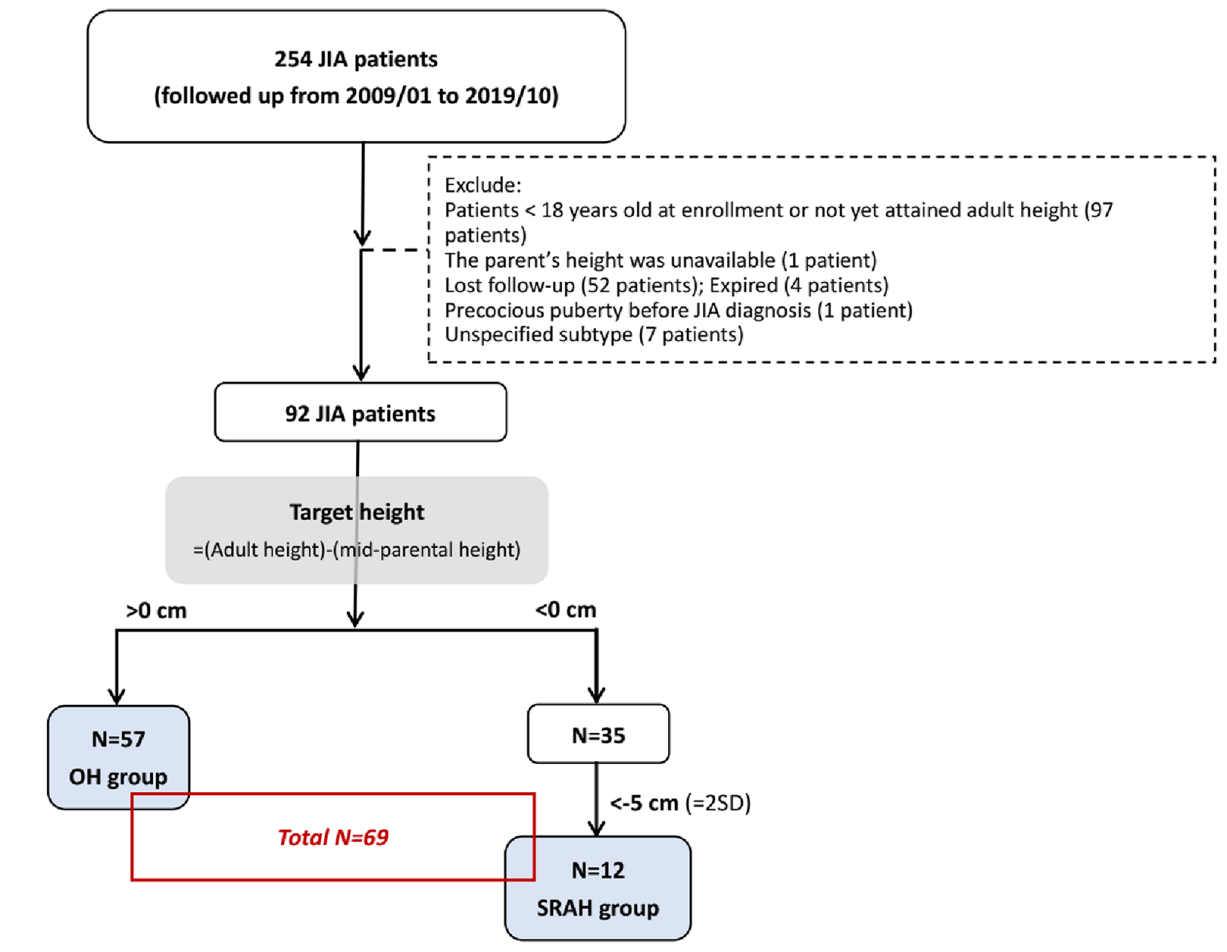
Flowchart of patient enrollment. JIA, juvenile idiopathic arthritis; AH, adult height, defined as current adult height; MPH, midparental height, defined as mean of parental height ± 6.5 cm; THD, target height difference, AH minus MPH; OH group, optimal height group, whose AH – MPH > 0 cm; SARH group, substantially reduced height group, whose AH – MPH <–5 cm; SD, standard deviation

We collected data from patients diagnosed with JIA between 2009 and 2019 at the National Taiwan University Children’s Hospital. JIA was diagnosed according to the International League of Associations for Rheumatology (ILAR) criteria [1]. Moreover, patients should be 18 years old upon enrollment and reach their AH while enrolled in our study. Those who were under regular follow-ups until disease remission were included. We excluded patients who belonged to the unspecified JIA type, lost follow-up, and died during the outpatient clinic follow-up until remission during the disease (Fig. 1).

### Measurement of height

At the clinic visit, we collected anthropometric data, including height (cm) and weight (kg). Height was measured by trained nurses using the same wall-mounted stadiometer. The parents’ heights were obtained at the clinic if available or from their self-measurements at home. We calculated the midparental height (MPH) by adding 6.5 cm to the mean of the parental height in males or by subtracting 6.5 cm in females. The differences between AH and MPH, the target height difference (THD) in this study, were calculated to minimize the inherent differences in each patient. Those patients with positive THDs belonged to the optimal height (OH) group. Patients whose THDs were below two standard deviations of the general population were defined as the substantially reduced height (SRAH) group. The definition of SRAH conformed to the clinical definition of short stature [17]. According to a previous study, the two standard deviations in the normal population were approximately 5 cm [18]. Significant factors for SRAH were compared and analyzed among the OH and SRAH groups.

### Baseline data collection

We reviewed each patient’s clinical characteristics, laboratory reports, therapeutic regimens, disease activity, and treatment response. The clinical features included age, sex, body mass index (BMI) at enrollment, age at disease onset, and JIA subtype. At initial diagnosis, laboratory data such as white blood cell count, hemoglobin level, platelet count, erythrocyte sedimentation rate (ESR), and C-reactive protein (CRP) level were recorded. Therapeutic regimens were collected and analyzed, such as disease-modifying antirheumatic drugs (DMARDs), methotrexate, systemic GC, and TNFα inhibitors. The administration route of systemic GC was oral prednisolone or intravenous methylprednisolone, with an equivalent accumulation dosage of prednisolone (mg/kg/day) recorded in cases who used GC for over a month. We also recorded the use of intra-articular corticosteroid injections (IACIs). The disease activity and treatment response were classified as active disease or remission (on/off medication), according to the preliminary criteria from Wallace 2004 [19]. Inactive disease is defined as the absence of clinical symptoms such as arthritis, arthralgia or uveitis, normal ESR, and CRP level, and no GC requirement at the last clinic visit. Remission on medication was based on the definition of inactive disease for more than six consecutive months, even under any treatment. Remission off medication was defined as an inactive disease status for at least 12 continuous months without any medication to treat arthritis or uveitis.

### Statistical analyses

For quantitative and ordinal data, patient data were expressed as counts, percentages, or medians with interquartile ranges (IQRs). Statistical analyses were performed using SAS software (version 9.4, SAS Institute Inc., Cary, USA). The Chi-square and Fisher’s exact tests were used to compare categorical variables. Differences between continuous variables were compared using an independent t-test. The Kruskal–Wallis rank test was used for nonparametric analysis within groups. Univariate and multiple logistic regression models were used to identify independent risk factors of SRAH development in JIA patients. We chose factors with a p-value less than 0.01 in the multiple logistic regression model with gender adjustment and checked the collinearity. Statistical significance was set at *p* < 0.05.

## Results

### Patients’ characteristics

**Fig. 2:**
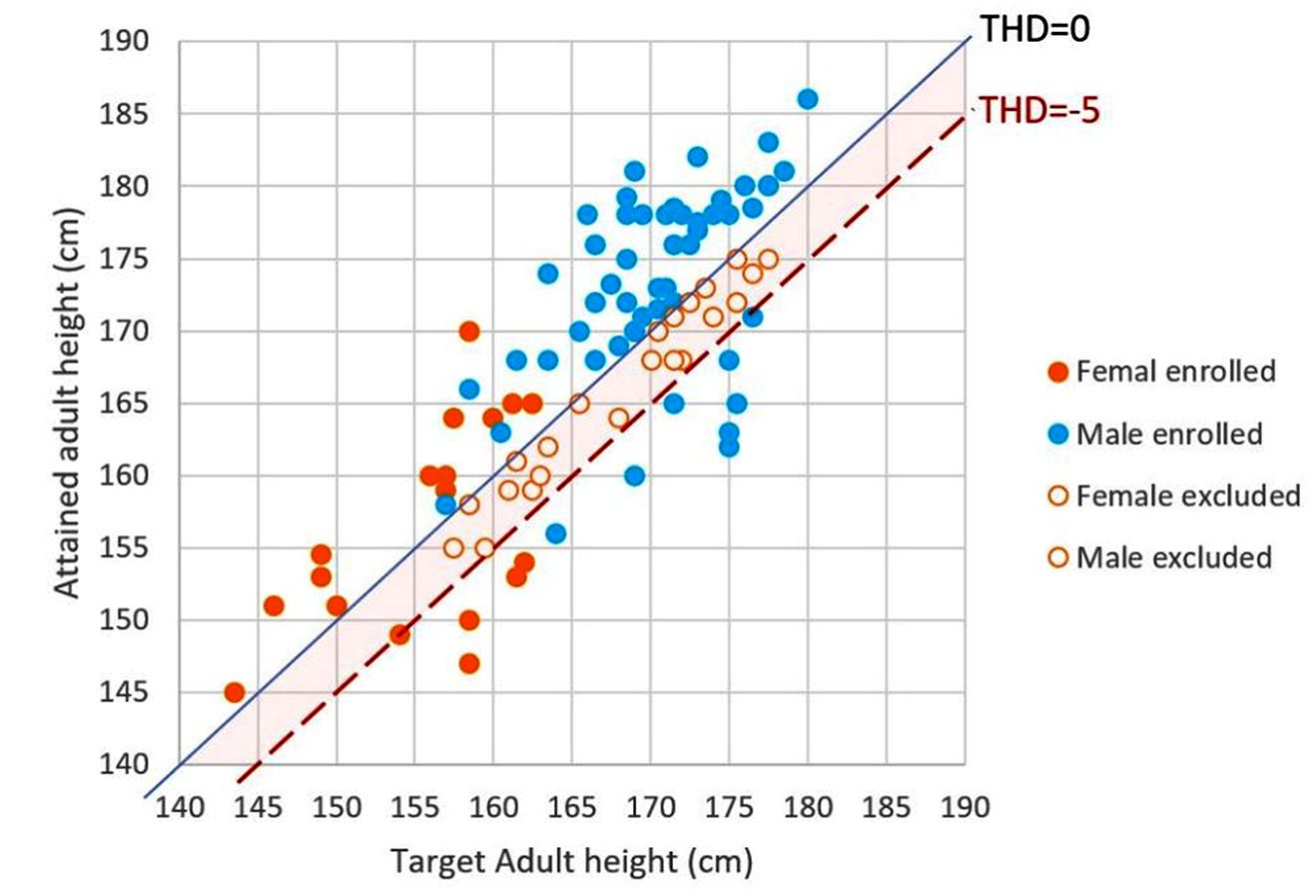
Distribution of JIA patients according to their adult height and midparental height (THD, target height difference)

A total of 92 patients were included in this study. The flowchart of patient enrollment is described in Fig. 1, and the height distribution based on their TH and MPD is in Fig. 2. We excluded those with THD between 0 cm and − 5 cm and enrolled the remaining 69 patients in the analysis, and there were 57 (82.6%) and 12 (17.4%) patients in the OH and SRAH groups, respectively. Their characteristics are listed in Table 1. The enrolled age was 22.2 years old in the OH group and 23.2 years old in the SRAH group. We observed a trend of earlier onset age in the SRAH group (9.6 vs. 11.7 years old, *p* = 0.20), especially before six years old, compared with the OH group (33.3% vs. 7.0%, *p* = 0.026). Males were predominant in both groups. The median (IQR) THD was 4.7 (3.1) cm in the OH group and − 9.0 (2.3) cm in the SRAH group. The distribution of JIA subtypes significantly differed between the two groups (*p* < 0.001). ERA was the most common subtype in the OH group, followed by the polyarticular type. However, the systemic type was the most common in the SRAH group. Initial laboratory data collected at the diagnosis were compared as listed in Table 1. No differences in other laboratory data were noted between the OH and SRAH groups, including antinuclear antibody (ANA), human leukocyte antigen (HLA)-B27, rheumatoid factor (RF) positivity, hemogram, or inflammatory markers.

**Table 1:** Patient characteristics in JIA patients with optimal height (OH) and substantially reduced height (SRAH)

	OH (*n* = 57)	SRAH (*n* = 12)	*p*-value
Male sex (n, %)	44 (77.2%)	8 (66.7%)	0.47
Enrolled age (years old)	22.2 (5.6)	23.2 (6.8)	0.52
The onset age of disease (years old)	11.7 (3.6)	9.6 (5.1)	0.20
The onset age of disease before 6 years old (n, %)	4 (7.0%)	4 (33.3%)	**0.026**
Final height (cm)	171.1 (9.1)	159.5 (7.5)	**< 0.001**
Final weight (kg)	64.1 (13.9)	55.1 (18.9)	**0.019**
Target height difference (cm)	4 0.7(3.1)	−9.0 (2.3)	**< 0.001**
BMI at enrolment (Kg/m^2^)	21.9 (4.1)	21.4 (6.2)	**0.211**
BMI classification (n, %)			0.51
Underweight	11 (20.0%)	4 (33.3%)	
Normal	37 (67.3%)	6 (50.0%)	
Overweight	4 (7.3%)	1 (8.3%)	
Obesity	3 (5.5%)	1 (8.3%)	
JIA classification (n, %)			**< 0.001**
Systemic	2 (3.5%)	5 (41.7%)	
Oligoarticular	10 (17.5%)	3 (25.0%)	
Polyarticular	11 (19.3%)	2 (16.7%)	
ERA	34 (59.6%)	2 (16.7%)	
History of uveitis after JIA diagnosed	4 (7.0%)	2 (16.7%)	0.28
**Lab data at diagnosis**			
Positive HLA-B27 (n, %)	33 (70.2%)	2 (33.3%)	0.16
Positive RF (n, %)	5 (9.1%)	0 (0%)	0.58
Positive ANA (n, %)	16 (28.1%)	3 (25.0%)	1.00
ESR (mm/h)	47.4 (42.5)	47.9 (40.7)	0.88
CRP (mg/dL)	3.3 (4.0)	3.5 (4.8)	0.85
WBC count (x10^3^/µL)	8449 (3733.4)	11,118 (6710.5)	0.08
Hb (g/L)	11.9 (2.1)	11.8 (1.7)	1.00
PLT count (x10^3^/µL)	358 (108)	418 (131)	0.15

Values are expressed as percentages (%) and mean with standard deviations

Target height difference (THD), adult height minus mid-parental height; BMI, body mass index; ERA, enthesis-related arthritis; HLA, human leukocyte antigen; RF, rheumatoid factor; ANA, antinuclear antibody; positive ANA, > 1:160(+); ESR, erythrocyte sedimentation rate; CRP, C-reactive protein; WBC, white blood cell count; Hb, haemoglobin; PLT, platelet

The treatment and disease statuses of enrolled patients are summarized in Table 2. In our study, GC remained one of the main treatments for active or severe disease activity. Around half of the patients in both groups had received GC for over one month. Most patients in both groups used GC for less than six months. Among the patients who used GC over one month, the average accumulation GC dose, equivalent to the prednisolone dosage, was 0.26 mg/kg/day, ranging from 0.01 to 0.77 mg/kg/day. Nine cases had received IACIs. Half the patients in the SRAH group had received GC for more than 12 months compared to the 23.3% in the OH group. All patients had received DMARDs, and methotrexate was frequently used. Though the difference did not reach significance, more patients in the SRAH group (91.7%) had received TNFα inhibitors and used them for a longer duration, with a mean time of 10.1 years, compared to the data in the OH group. As for the long-term treatment response and disease activity, more patients in the SRAH group still had an active disease status (50.0% vs. 21.1%, *p* = 0.066) at enrollment. Most patients (78.9%) in the OH group achieved inactive disease, 73.7% reached remission, and 17.5% did not take any medication. By comparison, in the SRAH group, 41.7% reached remission, but none were medication-free. Five patients had received orthopedic surgery due to complications of JIA. Four patients belonged to sJIA, and another one had ERA. Three were in the SRAH group, and the ratio was significantly higher than in the OH group (25% vs. 3.5%, *p* = 0.034).

**Table 2:** Treatments and disease course in JIA patients with optimal height (OH) and substantially reduced height (SRAH)

	OH group (*n* = 57)	SRAH group (*n* = 12)	*p*-value
Use of GC for more than one month (n, %)	30 (52.6%)	6 (50.0%)	1.00
Duration of GC treatment (months)	9.4 (14.5)	11.6 (12.0)	0.61
< 6 months (n, %)	18 (60.0%)	3 (50.0%)	0.45
6 to 12 months	5 (16.7%)	0 (0%)	
> 12 months	7 (23.3%)	3 (50.0%)	
Use of IACIs (n,%)	3 (5.3%)	1 (8.3%)	0.68
Use of MTX (n, %)	34 (59.6%)	7 (58.3%)	1.00
Number of DMARDs used (n, %)			0.92
One kind of DMARDs	16 (28.1%)	4 (33.3%)	
Two kinds of DMARDs	19 (33.3%)	4 (33.3%)	
Over two kinds of DMARDs	22 (38.6%)	4 (33.3%)	
Use of TNF-α inhibitors (n, %)	42 (73.7%)	11 (91.7%)	0.27
Age at TNF-α inhibitors onset (years old)	14.5 (4.3)	13.4 (5.6)	0.36
Time between diagnosis and treatment onset (years)	2.9 (3.8)	3.4 (6.9)	0.91
Duration of treatment (years)	8.4 (4.9)	10.1 (4.8)	0.14
Disease status (n, %)			0.066
Active disease	12 (21.1%)	6 (50.0%)	
Inactive disease without remission	3 (5.2%)	1 (8.3%)	
Inactive disease with remission	42 (73.7%)	5 (41.7%)	
Received orthopedic surgery due to JIA complications	2 (3.5%)	3 (25%)	**0.034**

Values are expressed as percentages (%) and mean with standard deviations

GC, glucocorticoids; IACIs, intra-articular corticosteroid injection; MTX, methotrexate; DMARDs, disease-modifying antirheumatic drugs; TNF-α, tumor necrosis factor

### Risk factors associated with SRAH in JIA patients

We conducted univariate logistic regression analyses to evaluate the risk factors of SRAH in JIA patients, as shown in Table 3. The onset age remained one of the factors of SRAH, especially in patients with earlier disease onset below six years old (OR = 6.63, 95%CI = 1.37–31.93, *p* = 0.018). The systemic subtype was significantly related to SRAH (OR = 42.5, 95%CI = 4.83–373.41, *p* = < 0.001), compared to the ERA subtype as the reference group. None of the laboratory data at the diagnosis and treatment course were factors related to SRAH. The patients with active disease status at enrollment had a significantly positive correlation with SRAH (OR = 4.2, 95%CI = 1.09–16.19, *p* = 0.037). History of orthopedic surgery due to complications of JIA was significantly related to SRAH (OR = 9.17, 95%CI = 1.34–62.71, *p* = 0.024). After adjustment, sJIA remained the risk factor of SRAH (OR = 37.6, 95%CI 1.2-1210.5; *p* = 0.041).

**Table 3:** Univariate and multiple logistic regression analyses for factors affecting SRAH

Variables	Univariate regression		Multiple regression	
	OR (95%CI)	*P*-value	Adjusted OR (95%CI)	*p*-value
Male sex	0.59 (0.15, 2.28)	0.445		
Final weight	0.95 (0.90,1.00)	**0.067**	0.99 (0.93, 1.04)	0.61
BMI at enrolment	0.98 (0.84,1.13)	0.74		
JIA onset age	0.88 (0.75, 1.02)	0.09		
JIA onset age before 6 years old	6.63 (1.37, 31.93)	**0.018**	2.14 (0.16, 28.9)	0.57
**JIA subtype**				
ERA	1		1	
Systemic JIA	42.5 (4.83, 373.41)	**< 0.001**	37.6 (1.17, 1210.5)	**0.041**
Oligoarticular JIA	5.1 (0.75, 34.89)	0.097	3.46 (0.38, 31.13)	0.27
Polyarticular JIA	3.1 (0.39, 24.61)	0.29	2.14 (0.23, 19.57)	0.50
History of uveitis	0.30 (0.43,16.47)	0.30		
**Lab data at diagnosis**				
ESR (mm/h)	1.00 (0.98,1.09)	0.96		
CRP (mg/dL)	1.02 (0.88,1.18)	0.84		
WBC count (x10^3^/µL)	1.00 (1.00,1.00)	0.10		
Hb (g/L)	0.98 (0.72,1.35)	0.92		
PLT count (x10^3^/µL)	1.00 (0.99,1.01)	0.11		
Duration of GC treatment	1.01 (0.96, 1.06)	0.93		
Use of MTX	0.95 (0.27, 3.35)	0.93		
Use of TNF-α inhibitor	0.21 (0.47, 33.07)	0.21		
Age at TNF-α inhibitor use	0.95 (0.82, 1.11)	0.53		
Time from diagnosis to TNF-α inhibitor use	1.03 (0.89, 1.20)	0.71		
Duration of TNF-α inhibitor use (years)	1.07 (0.94, 1.22)	0.31		
**Disease status**				
Inactive disease with remission	1		1	
Inactive disease without remission	2.8 (0.24, 32.31)	0.41	3.91 (0.25, 61.38 )	0.33
Active disease	4.2 (1.09, 16.19)	**0.037**	4.12 (0.66, 2.52)	0.13
History of orthopedic surgery due to JIA	9.17 (1.34, 62.71)	**0.024**	0.34 (0.005, 25.63)	0.63

SRAH, substantially reduced adult height; BMI, body mass index; JIA, juvenile idiopathic arthritis; GC, glucocorticoids; MTX, methotrexate; ESR, erythrocyte sedimentation rate; CRP, C-reactive protein; WBC, white blood cell count; Hb, hemoglobin; PLT, platelet; TNF-α, tumor necrosis factor; inactive disease is defined as the absence of symptoms and normal ESR/CRP level and no requirement of GC at the time of enrolment

### Subgroup analysis by gender

To eliminate the effects on growth in different genders, we performed a subgroup analysis according to the gender of each patient (Supplementary Table 1). Among the 52 male patients, 44 were in the OH group and 8 in the SRAH group. The JIA subtypes showed significant differences between the OH and SRAH groups (*p* = 0.025). ERA was the most common subtype in the OH group (65.9%); however, all subtypes were evenly distributed in the SRAH group. Besides, the disease status in both groups differed (*p* = 0.038), with most of the OH group achieving remission status (79.5%) and half of the patients in the SRAH group still in active disease.

There were 17 female patients, including 13 in the OH group and 4 in the SRAH group. The patients in the SRAH group had significantly earlier disease onset age than those in the OH group (4.3 vs. 12.0 years old, *p* = 0.032). A significantly lower BMI was found in the SRAH group (17.9$\pm$1.8 vs. 21.9$\pm$4.4 kg/m2, *p*=0.015). The distribution of the JIA subtype in female patients showed similar results in the total population, with more ERA (38.5%) and polyarticular (38.5%) subtypes in the OH group and primarily systemic (75%) subtype in the SRAH group (*p* = 0.016). Two initial laboratory parameters, ESR and WBC count, were significantly higher in the SRAH group. Also, patients in the SRAH group required longer treatment durations of TNFα inhibitors (15.1$\pm$1.6 vs. 6.3$\pm$7.9 years, *p* = 0.049).

## Discussion

In this single-center cohort study, we analyzed the factors affecting JIA patients’ final AH, including basic characteristics, laboratory reports, treatments, and disease status. We found sJIA subtype, an earlier disease onset, mainly before six years old, a history of JIA-associated orthopedic surgery, and active disease status during follow-ups were correlated with SRAH in JIA patients. This study may be the first one to discuss SRAH in JIA patients by comparing the final and estimated AH based on the MPH.

Since chronic inflammation, the essential feature of JIA, is often related to growth disorders ranging from a mild decrease in growth velocity to severe growth impairment, many studies assessed the growth in JIA patients and the factors associated with growth restriction or short stature. The previous studies regarding growth in JIA used z-scores as a height parameter and for the definition of short stature compared to the general population. Our study used the THD based on the patient’s MPH to minimize the genetic effects of their parent’s height. We reported that 13.0% of JIA patients had SRAH, with 71.4% in children with sJIA, 23.1% in children with the oligoarticular disease, 15.3% in children with the polyarticular disease, and only 0.5% in children with ERA. Because ERA remains the predominant JIA subtype in Taiwan based on previous epidemiologic studies, the rate of SRAH is relatively lower in our JIA patients [20].

Our study found that patients with sJIA accounted for most of the SRAH group (41.7%), and sJIA was the most essential predicting factor for SRAH in our results. McErlane et al. reported that patients with sJIA had higher rates of severe growth restriction and lower height growth velocity among all JIA subtypes [21]. Barut et al. reported a 15-year and single-center experience of the clinical features and outcomes in 168 sJIA patients. Growth retardation was recorded in 11.3% of them, especially in those patients with persistent or polycyclic clinical courses [22]. Systemic JIA was characterized by its significant systemic inflammation, earlier disease onset, longer disease course, and poor disease outcome [23]. Several studies supported evidence that the inflammatory process may promote growth hormone resistance and disease [8, 11]. The findings of association between persistent inflammation and growth indicate that sJIA patients may experience reduced height in adulthood than other subtypes due to profound inflammatory processes in this group of patients.

A variety of drugs have been found to affect the AH in JIA. Glucocorticoids, useful anti-inflammatory drugs, were often used in JIA patients with flared-up diseases and were usually prescribed a few decades ago. Wang et al. reported the negative influence of GC on AH in JIA patients, according to the data collected from 1973 to 1995 at the same medical institute in this study [14]. However, we did not find significant differences between the OH and SRAH groups regarding the duration of GC use. The main difference may be that 79.3% of the JIA patients enrolled were diagnosed after 2003, when the first biologic for JIA was introduced in our hospital, and patients had more treatment options like DMARDs than GC. Only a few studies have reported a correlation between DMARDs and final AH. An open-label, nonrandomized trial in 2010 showed a significant increase in the mean height percentiles when methotrexate (MTX) and Etanercept were combined in polyarticular or sJIA after three years of follow-up [24]. This report suggested that multiple medication use may improve the inflammatory process and indirectly decrease body height. Further subgroup analysis may be required to demonstrate the effect of treatment in different JIA subgroups.

In our study, orthopedic surgery was significantly related to SRAH. Previous cohort studies showed that 10% of patients with JIA needed orthopedic surgeries, such as total hip and knee arthroplasty, to correct their bone deformity or joint contracture [25, 26]. Our study found that all JIA patients with SRAH and previous operation history had an active disease condition at enrollment. This result informed us that those with active disease and poor treatment response might require orthopedic surgery, and these populations may also experience short AH due to their severe disease status.

This study had some limitations. First, this was a retrospective cohort study with a recall and selection bias. However, we enrolled those patients with continuous follow-ups and collected the primary outcomes objectively to minimize bias. Second, our study lacked serial disease severity scores to clarify the effect of long-term disease severity on AH. However, we used the latest disease status to understand the relationships between JIA disease control and SRAH. Lastly, the case number was insufficient to perform a subgroup analysis of the JIA subtypes. The data presented here could provide important information about AH in the post-biologics era of JIA. However, more detailed prospective records and evaluations of different JIA subtypes are still needed in the future.

## Conclusion

Reduced AH is one of the main concerns in patients with JIA. In this real-world experience, we identified factors such as systemic subtype, younger disease onset age, active disease status, and history of JIA-related orthopedic surgery that were correlated with SRAH in patients with JIA. Among them, sJIA was the main factor affecting AH. For JIA patients with these characteristics, clinicians might need to be aware of their growth problems during the disease course and provide intervention if signs of growth retardation present.

### Electronic supplementary material

Below is the link to the electronic supplementary material.

Supplementary Material 1

## Data Availability

All data generated or analyzed during this study were included in this manuscript.

## Abbreviations

AH: Adult height
ANA: Antinuclear antibody
BMI: Body mass index
CRP: C-reactive protein
DMARDs: Disease-modifying antirheumatic drugs
ERA: Enthesis-related arthritis
ESR: Erythrocyte sedimentation rate
HLA: Human leukocyte antigen
ILAR: International League of Associations for Rheumatology
JIA: Juvenile idiopathic arthritis
OH: Optimal height
MPH: Midparental height
RH: Reduced height
SRAH: Substantially reduced adult height
THD: Target height difference
TNF: Tumor necrosis factor
