# Supplementary material for: Identifying factors associated with substantially reduced adult height in patients with juvenile idiopathic arthritis: a retrospective cohort study
Source: BMC Pediatr. 2024 May 30;24:375. doi: 10.1186/s12887-024-04855-3 (PMC11137984; doi:10.1186/s12887-024-04855-3)
Supplement: Supplementary file 1 — Supplementary Material 1 [file 12887_2024_4855_MOESM1_ESM.docx]

**Supplementary table 1**. Patient characteristics in male/female patients with optimal height (OH) and substantially reduced height (SRAH).

|  |  | Male |  |  | Female |  |
| --- | --- | --- | --- | --- | --- | --- |
|  | OH  (n=44) | SRAH  (n=8) | p-value | OH  (n=13) | SRAH  (n=4) | p-value |
| Enrolled age (year-old) | 22.1 (5.9) | 21.3 (4.2) | 0.44 | 22.5 (5.6) | 21.5 (7.0) | 0.955 |
| Onset age of disease (years old) | 12.6 (4.7) | 12.8 (4.1) | 0.90 | 12.1 (6.0) | 4.3 (4.1) | **0.032** |
| Onset age before 6-year-old | 3 (6.8%) | 1 (12.5%) | 050 | 1 (7.7%) | 3 (75.0%) | **0.022** |
| Final height (cm) | 176.0 (7.1) | 164.0 (4.3) | **<.001** | 160 (4.0) | 152 (11.0) | 0.069 |
| Target height difference (cm) | 4.5 (4.1) | -8.5 (4.0) | **<.001** | 4.0 (2.5) | -8.5 (0.9) | **0.004** |
| Final weight (Kg) | 64.5 (18.0) | 56.5 (13.0) | 0.28 | 55.0 (15.0) | 40.5 (1.5) | **0.004** |
| BMI (Kg/m^2^) | 20.9 (4.8) | 20.6 (5.3) | 0.89 | 21.9 (4.4) | 17.9 (1.8) | **0.015** |
| BMI classification (n, %) |  |  | 0.45 |  |  | 0.73 |
| Underweight | 9 (21.4%) | 2 (25%) |  | 2 (15.4%) | 2 (50%) |  |
| Normal | 28 (66.7%) | 4 (50%) |  | 9 (69/2%) | 2 (50%) |  |
| Overweight | 3 (7.1%) | 1 (12.5%) |  | 1 (7.7%) | 0 (0%) |  |
| Obesity | 2 (4.8%) | 1 (12.5%) |  | 1 (7.7%) | 0 (0%) |  |
| JIA classification (n, %) |  |  | **0.025** |  |  | **0.016** |
| Systemic | 1 (2.3%) | 2 (25.0%) |  | 1 (7.7%) | 3 (75%) |  |
| Oligoarticular | 8 (18.2%) | 2 (25.0%) |  | 2 (15.4%) | 1 (25%) |  |
| Polyarticular | 6 (13.6%) | 2 (25.0%) |  | 5 (38.5%) | 0 (0%) |  |
| ERA | 29 (65.9%) | 2 (25.0%) |  | 5 (38.5%) | 0 (0%) |  |
| History of uveitis after JIA diagnosed | 4 (9.1%) | 2 (25.0%) | 0.23 | 0 (0%) | 0 (0%) | - |
| Lab data at diagnosis |  |  |  |  |  |  |
| Positive HLA-B27(n, %) | 28 (71.8%) | 2 (33.3%) | 0.16 | 5 (62.5%) | 0 (0%) | - |
| Positive RF (n, %) | 2 (4.8%) | 0 (0%) | 1.00 | 0 (0%) | 3 (23.1%) | 0.54 |
| Positive ANA (n, %) | 10 (22.7%) | 2 (25.0%) | 1.00 | 6 (46.2%) | 1 (25 %) | 0.60 |
| ESR (mm/h) | 51.0 (57.5) | 18.5 (51.0) | 0.10 | 27.5 (18.8) | 87.0 (56.3) | **0.028** |
| CRP (mg/dL) | 2.0 (3.3) | 0.8 (1.2) | 0.20 | 0.7 (3.3) | 5.8 (3.8) | 0.30 |
| WBC count (x10^3^/μL) | 8845 (3023) | 9875 (3363) | 0.26 | 7000 (990) | 9040 (2130) | **0.045** |
| Hb (g/L) | 11.9 (2.2) | 12.9 (1.1) | 0.25 | 11.5 (2.0) | 10.3 (1.4) | 0.098 |
| PLT count (x10^3^/μL) | 358 (157) | 420 (96.3) | 0.40 | 333 (109) | 472 (246) | 0.257 |

Values are expressed as percentages (%) and median with interquartile range (IQR).

Target height difference (THD), adult height minus mid-parental height; BMI, body mass index; ERA, enthesis-related arthritis; HLA, human leukocyte antigen; RF, rheumatoid factor; ANA, antinuclear antibody; positive ANA, >1:160(+); ESR, erythrocyte sedimentation rate; CRP, C-reactive protein; WBC, white blood cell count; Hb, hemoglobin; PLT, platelet

|  |  | Male |  |  | Female |  |
| --- | --- | --- | --- | --- | --- | --- |
|  | OH  (n=44) | SRAH  (n=8) | p-value | OH  (n=13) | SRAH  (n=4) | p-value |
| Use of GC for more than one month (n, %) | 24 (54.5%) | 4 (50%) | 1.00 | 6 (46.2%) | 2 (50%) | 1.00 |
| Duration of GC treatment (months) | 0.2 (5.0) | 1.4 (3.3) | 0.94 | 0.0 (3.4) | 8.0 (18.7) | 0.43 |
| Use of MTX (n, %) | 26 (59.1 %) | 5 (62.5%) | 1.00 | 8 (61.5%) | 2 (50%) | 1.00 |
| Number of DMARDs used (n, %) |  |  | 0.78 |  |  | 0.29 |
| one kind of DMARDs | 12 (27.3%) | 2 (25%) |  | 4 (30.8%) | 2 (50%) |  |
| two kinds of DMARDs | 13 (29.5%) | 4 (50%) |  | 6 (46.2%) | 0 (0%) |  |
| Over two kinds of DMARDs | 19 (43.2%) | 2 (25%) |  | 3 (23.0%) | 2 (50%) |  |
| Use of TNF-α inhibitors (n, %) | 32 (72.7%) | 8 (100%) | 0.18 | 10 (76.9%) | 3 (75.0%) | 1.00 |
| Age at TNF-α inhibitors onset (years old) | 14.6 (4.8) | 13.8 (4.8) | 0.81 | 15.8 (0.8) | 5.9 (12.8) | 0.45 |
| Time between diagnosis and treatment onset (years) | 1.1 (3.1) | 0.9 (1.2) | 0.50 | 3.2 (6.0) | 2.4 (8.8) | 0.69 |
| Duration of treatment (years) | 8.2 (6.1) | 8.9 (6.8) | 0.76 | 6.3 (7.9) | 15.1 (1.6) | **0.049** |
| Disease status (n, %) |  |  |  |  |  |  |
| Active disease | 7 (15.9%) | 4 (50%) | **0.038** | 5 (38.5%) | 2 (50%) | 1.00 |
| Inactive disease without remission | 2 (4.5%) | 1 (12.5%) |  | 1 (7.7%) | 0 (0%) |  |
| Inactive disease with remission | 35 (79.5%) | 3 (37.5%) |  | 7 (53.8%) | 2 (50%) |  |
| Received orthopedic surgery due to JIA complications | 1 (2.3%) | 1 (12.5%) | 0.29 | 1 (7.7%) | 2 (50%) | 0.12 |

**Supplementary table 2**. Treatments and disease course in JIA patients with optimal height (OH) and substantially reduced height (SRAH).

Values are expressed as percentages (%) and mean with standard deviations
GC, glucocorticoids; MTX, methotrexate; DMARDs, disease-modifying antirheumatic drugs; TNF-α, tumour necrosis factor; inactive disease is defined as absence of symptoms and normal ESR/CRP level and no requirement of steroid at the moment of enrolment; remission on medication is defined as inactive disease for more than 6 consecutive months and was under medication; remission off medication was defined as inactive disease for more than 12 consecutive months and was without medication
